# Perturbation of gut microbiota decreases susceptibility but does not modulate ongoing autoimmune neurological disease

**DOI:** 10.1186/s12974-020-01766-9

**Published:** 2020-03-06

**Authors:** Clemens Gödel, Birgit Kunkel, Alireza Kashani, Hans Lassmann, Manimozhiyan Arumugam, Gurumoorthy Krishnamoorthy

**Affiliations:** 1 Department of Neuroimmunology, Max Planck Institute of Neurobiology, Martinsried, Germany; 2 Faculty of Medicine, University of Freiburg, Freiburg, Germany; 3 Research group Neuroinflammation and mucosal Immunology, Max Planck Institute of Biochemistry, Martinsried, Germany; 4 Novo Nordisk Foundation Center for Basic Metabolic Research, Faculty of Health and Medical Sciences, University of Copenhagen, Copenhagen, Denmark; 5 Department of Neuroimmunology, Medical University of Vienna, Vienna, Austria

**Keywords:** Multiple sclerosis, EAE, Microbiota, Antibiotics, CNS autoimmunity, microbiome

## Abstract

The gut microbiota regulates the host immune and nervous systems and plays an important role in the pathogenesis of autoimmune neurological disease multiple sclerosis (MS). There are considerable efforts currently being undertaken to develop therapies for MS based on the modulation of microbiota. Evidence from experimental models suggests that the manipulation of microbiota through diet or antibiotics prior to the disease development limits disease susceptibility. However, it is currently unclear if microbiota manipulation therapies would also have an impact on ongoing neurological disease. Here, we examined the effect of antibiotic-based microbiota modulation in spontaneous experimental autoimmune encephalomyelitis (EAE) mouse models of MS before and after the onset of autoimmune disease. Prophylactic antibiotic treatment led to a significant reduction of susceptibility to spontaneous EAE. In contrast, antibiotic treatment after the onset of spontaneous EAE did not show a significant amelioration. These results reveal that the perturbation of gut bacteria alters disease susceptibility but has minimal impact on the ongoing neurological disease.

## Introduction

Advances in DNA sequencing and bioinformatics technologies in the last two decades led to an exponential increase of studies that investigated the role of the microbiome, in particular, gut microbiome, in several diseases. This surge in interest has specifically garnered attention in clinical settings due to their potential applications in clinical practice [1, 2]. The importance of microbiota in specific diseases has been shown predominantly in animal models or by comparing the community structure of the gut microbiota in patients compared to controls. Most of the results that have shown a causal relationship with the microbiota to the disease pathogenic processes have used germ-free (GF) mice or manipulation of microbiota prior to the disease development. Based on this, it is assumed that gut microbiota manipulation would have therapeutic benefits. However, the relative importance of microbiota in already ongoing diseases is unclear.

We and others have previously shown that gut microbiota is essential for the central nervous system (CNS)-specific autoimmune disease, multiple sclerosis (MS) [3–5]. In animal models of MS, manipulation of microbiota prior to the development of the disease either by antibiotics [6–8] or diet [9–11] modulated disease severity and incidence. However, it is unclear if the modulation of microbiota in ongoing disease would also have an impact on the disease progression. Here, using spontaneous EAE mouse models of MS, we studied the relative importance of the microbiota before and after the development of the neurological disease.

## Methods

### Mice, antibiotic treatment, and scoring

OSE (2D2 × IgH^MOG^) C57BL/6 [12] and RR SJL/J [13] mice were bred and housed at the animal facility of the Max Planck Institute of Biochemistry, Martinsried. Mice were treated with ampicillin (1 g/L), neomycin (1 g/L), and vancomycin (1 g/L) dissolved in autoclaved drinking water. To increase the liquid intake, sweetener was added and the control mice also received the same sweetener in their drinking water. Since the disease development in the OSE model starts as early as 5 weeks after birth [12], OSE mice were given antibiotics either at 2 or 4 weeks of age in prophylactic treatment experiments. For therapeutic treatment experiments, mice that developed spontaneous EAE symptoms (after first signs of EAE) were randomly assigned to either control or antibiotic group and treated for 2 weeks as above. Clinical disease was assessed according to the standard 5-point scale [12, 13]. All animal procedures were approved by Regierung von Oberbayern (Munich, Germany).

### Histology

Animals were sacrificed 2 weeks after treatment. The brain and spinal cords were fixed in formaldehyde and embedded in paraffin. The neuropathological analysis was done on paraffin sections of the brain (including optic nerve and chiasm) and the spinal cord stained with hematoxylin/eosin for inflammation and Luxol fast blue myelin stain for demyelination. Semiquantitative assessment of the degree of inflammation and demyelination was performed in the spinal cord and the optic nerves. The entire spinal cord was embedded in 10 standardized tissue blocks. Inflammation was quantified by counting the number of perivenous inflammatory infiltrates and providing their average number per spinal cord cross section per animal. The extent of demyelination in the spinal cord was evaluated according to the following scoring: 0, no demyelination; 1, perivenous and subpial demyelination; 2, confluent demyelinated plaques; and 3, extensive demyelination affecting more than half of the spinal cord cross section. The extent of demyelination in the optic nerve was evaluated according to the following scoring: 0, no demyelination; 1, perivenous demyelination; 2, perivenous and subpial demyelination; 3, confluent demyelinated plaques; and 4, complete focal demyelination in the optic nerve. Representative pictures for the demyelination scores are given in Supplementary Figure 2.

### Cell isolation and flow cytometry

Isolation and phenotyping of immune cells by flow cytometry were done as previously described [4]. For the isolation of lymphocytes from the small intestine, the intestine was collected in ice-cold Hank’s Balanced Salt Solution (HBSS) buffered with 15 mM Hepes. After careful removal of Peyer’s patches, fatty tissue, and fecal contents, the intestine was opened longitudinally and cut into small pieces. The intestinal fragments were washed three times for 15 min in HBSS containing 5 mM EDTA, 15 mM Hepes, and 10% fetal bovine serum (FBS). Next, intestinal pieces were washed once for 5 min with stirring in Roswell Park Memorial Institute medium (RPMI) containing 15 mM HEPES and 10% FBS, followed by an incubation step at 37 °C with stirring (550 rpm) in RPMI with 15 mM HEPES, 10% FBS, and 100 U/ml collagenase D (Roche Diagnostics). The digested tissue was washed twice in HBSS containing 5 mM EDTA, before the lymphocytes of the small intestine were resuspended in 5 ml of 40% Percoll (Sigma-Aldrich) and overlaid on 2.5 ml of 80% Percoll. Percoll gradient separation was performed by centrifugation at 780*g* for 20 min at room temperature. Small intestinal lamina propria lymphocytes were harvested from the interphase of the Percoll gradient and washed once in RPMI containing 15 mM Hepes and 10% FBS. After isolation, cells were stained in FACS buffer (PBS containing 1% BSA and 0.1% NaN_3_) with fluorochrome-labeled Abs against CD45 (30-F11), CD4 (RM4-5), B220 (RA3-6B2), CD11b (M1/70), and CD11c (N418). For intracellular cytokine staining, cells were activated with 50 ng/ml PMA (Sigma-Aldrich) and 500 ng/ml ionomycin (Sigma-Aldrich) in the presence of 5 μg/ml brefeldin A (Sigma-Aldrich) for 4 h at 37 °C. After surface staining, cells were fixed and permeabilized using the Transcription Factor Staining Buffer Set (eBioscience) and stained intracellularly using the following antibodies against IFN-γ (XMG1.2), IL17 (TC11-18H10), IL-22 (1H8PWSR), and TNFα (MP6-XT22). All antibodies were purchased from BD Pharmingen, eBioscience, or BioLegend. Cells were acquired on FACSVerse, and analysis was performed using FlowJo (TreeStar) software.

### Culture of bacteria

Fecal pellets were collected from the mice and frozen at − 80 °C until analysis. Fecal pellets of mice were weighed, suspended, and serially diluted in sterile PBS. One hundred microliters of the diluted fecal homogenates were plated on Bile Esculin (BE) agar plates. After overnight incubation at 37 °C, the colony-forming units (c.f.u.) were determined and expressed as c.f.u./g of feces.

### Bacterial DNA extraction from mouse feces and 16S RNA sequencing

Fecal pellets were collected from the mice and frozen at − 80 °C until analysis. Bacterial genomic DNA was extracted from fecal pellets using QIAamp DNA Stool mini kit with bead beating (Qiagen). An amplicon library targeting V3 and V4 regions of the 16S ribosomal RNA gene was amplified by PCR with specific primers and sequenced on an Illumina MiSeq instrument (2 × 300-bp paired-end reads). Paired reads were merged to assemble a contig within a length of 456 ± 11 base pairs. Contigs with low base pair quality scores as well as those with misprimes were removed. Sequences were assigned to the same species-level OTU if they had a 97% sequence identity. To identify differentially abundant members of the gut microbiome between the groups, we performed a Wilcoxon rank-sum test on OTU relative abundances and adjusted the *P* values for multiple testing using the false discovery rate (FDR). OTUs with an adjusted *P* value less than 0.05 were considered as differentially abundant.

### Quantitative PCR for bacteria

Bacterial genomic DNA was extracted from fecal pellets using the QIAamp DNA Stool mini kit (Qiagen). Total or enterococci 16S rRNA gene-specific PCR was performed. Primers for enterococci 16S rRNA gene were as follows: GTGCCAGCMGCCGCGGTAA and GCCTCAAGGGCACAACCTCCAAG. Primers for total 16S rRNA gene were as follows: ACTCCTACGGGAGGCAGCAGT and ATTACCGCGGCTGCTGGC. All reactions were performed using the SYBR Green qPCR master Mix (Thermo Fisher Scientific) following the manufacturer’s instructions and measured using a 7900HT real-time PCR System or Quantstudio 3 (Applied Biosystems). The absolute quantity of 16S rRNA gene copies was calculated using a standard curve generated with a plasmid encoding enterococci or lactobacilli (for total 16S rRNA) 16S rRNA gene and expressed as gene copies per nanogram of DNA.

### Statistics

GraphPad Prism 6 (GraphPad Software, Inc.) was used for the statistical analysis. *P* values < 0.05 were considered to be significant.

## Results and discussion

**Fig. 1:**
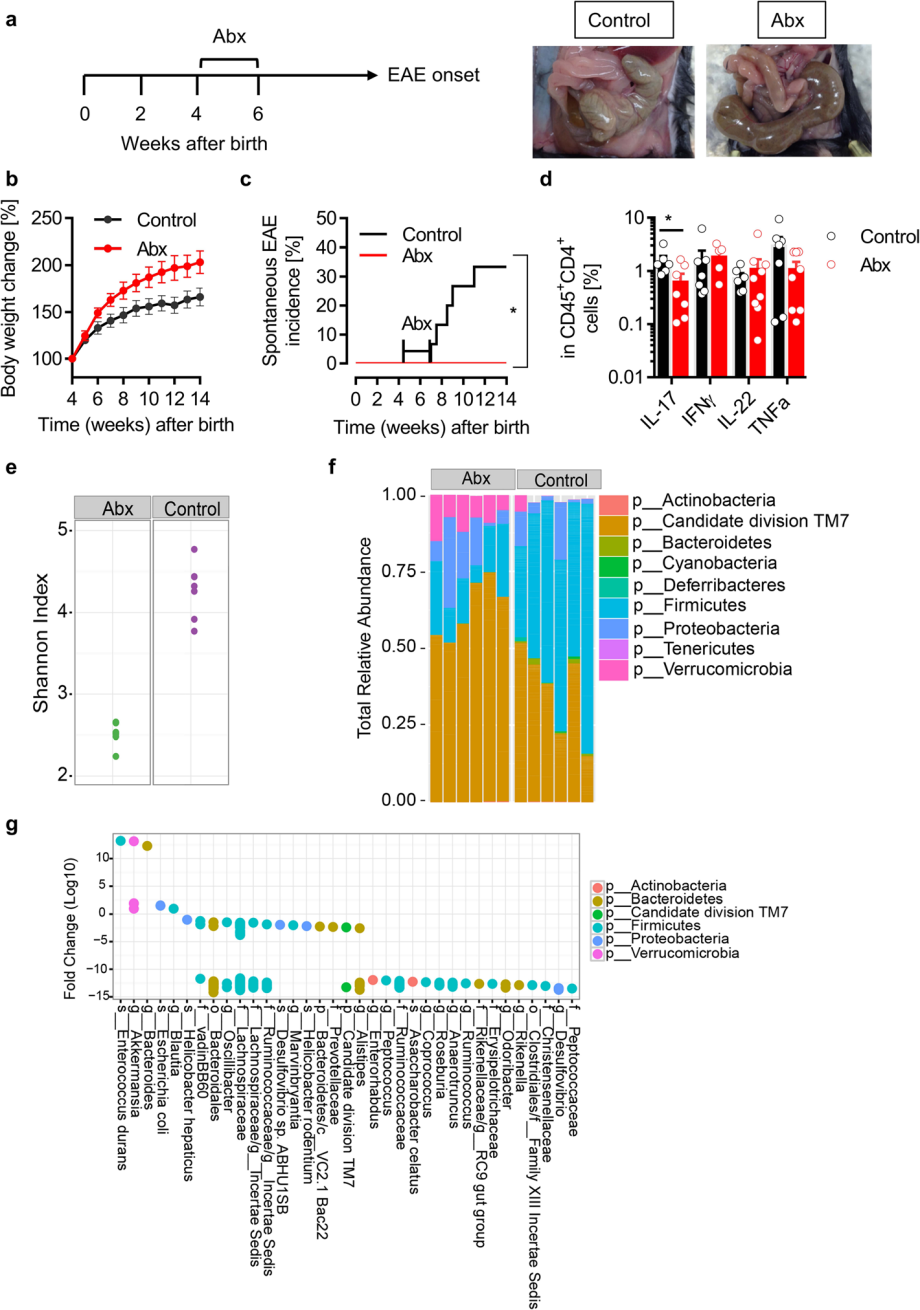
Antibiotic treatment prevents spontaneous CNS autoimmune disease. **a** Schematic view of the treatment protocol. Significant increase in the cecum size following antibiotic treatment. Exemplary pictures are shown. **b** Bodyweight change in percent compared to the initial weight. *n* = 10 per group. **c** Incidence of spontaneous EAE in a cohort of antibiotic-treated OSE mice. *n* = 15 per group. *P < 0.05 (Gehan-Breslow-Wilcoxon test). **d** Frequencies of IL-17, IFNγ, IL-22, and TNF-α-producing CD4^+^CD45^+^cells in the small intestine from control and antibiotic-treated mice. *n* = 6–8 mice per group. *P < 0.05 (Mann-Whitney test). **e** Comparison of fecal microbiome alpha-diversities (Shannon index) of control and antibiotic-treated mice. **f** Comparison of fecal microbiome phylum compositions in control and antibiotic-treated mice. **g** Fold changes of significant OTUs identified by the Wilcoxon rank-sum test as differentially abundant in fecal microbiomes. OTUs are colored by phylum affiliation and grouped by the highest taxonomic resolution

Previous studies have shown that manipulation of microbiota with prophylactic antibiotic treatment suppressed the severity of autoimmune encephalomyelitis in actively induced EAE models [6–8]. To avoid potential influence of the adjuvant CFA (commonly used in active EAE models) in treatment-related immune responses, we treated a spontaneous experimental autoimmune encephalomyelitis mouse model (OSE (opticospinal encephalomyelitis)) [12] with a mixture of antibiotics before the development of the disease. OSE mice were given an antibiotic mixture for 2 weeks starting from either 2 or 4 weeks after birth (Fig. 1 a). After 2 weeks of antibiotic treatment, mice were given access to normal tap water. The effect of antibiotic treatment was clearly visible in the cecum size. The antibiotic-treated mice had significantly larger cecum than control mice (Fig. 1c). Also, antibiotic-treated mice had a slight increase in body weight when compared to control mice (Fig. 1b). Similar to previous reports in actively induced EAE models [6–8], prophylactic antibiotic treatment either at 2 or 4 weeks after birth suppressed the development of spontaneous EAE to a greater extent (Fig. 1c and data not shown). Apart from antibiotics, we have previously shown that prophylactic dietary fiber supplementation which alters gut microbiota also prevented the development of spontaneous EAE by modulating microbiota [9]. Analysis of intestinal immune cells populations showed no changes in the frequencies of CD4^+^ T cells, B220^+^ B cells, CD11b^+^ macrophages, and CD11c^+^ dendritic cells. However, there was a decrease in the frequencies of proinflammatory IL-17- and TNFα-producing T cells while IFNγ- and IL-22-producing T cells were unaltered (Fig. 1d).

Since antibiotics can alter the composition of the microbiota, we analyzed the fecal microbiota by 16S ribosomal RNA (16S rRNA) gene amplicon sequencing. Gut bacterial communities between control and antibiotic-treated mice were compared in terms of diversity (alpha and beta) and composition. The beta diversity analysis revealed distinct clustering of control and antibiotic-treated mice, suggesting that antibiotics significantly altered the overall bacterial community composition. The alpha diversity analysis revealed that species diversity (Shannon index) is significantly reduced in the antibiotic-treated mice (Fig. 1e). The analysis of the abundance profiles of bacterial populations showed various alterations in the microbiota of antibiotic-treated mice (Fig. 1f). While many of the significantly altered microbial organisms were depleted in antibiotic-treated mice, we found an increased representation of *Enterococcus durans* species together with *E*. *coli* and other species within the genera *Akkermansia, Bacteroides*, and *Blautia* in antibiotic-treated mice (Fig. 1g). We confirmed the increased abundance of the enterococci by quantitative PCR using bacterial DNA isolated from fecal pellets and by culture on the Bile Esculin agar plate, which allows the selective growth of *Enterococcus* species (Supplementary Fig. 1). *Enterococcus durans* has been shown to have anti-inflammatory properties. It reduced the expression of proinflammatory cytokines IL-1β and IL-17A and increased the proportion of regulatory T cells in a DSS colitis model, which resulted in the improvement of clinical scores and pathology [14]. However, our attempts to stably colonize enterococci in our OSE mice were not successful likely due to the colonization resistance by the indigenous bacteria. It is plausible that *E*. *durans* along with other bacterial strains that are enriched after antibiotic treatment induces anti-inflammatory responses in our model.

**Fig. 2:**
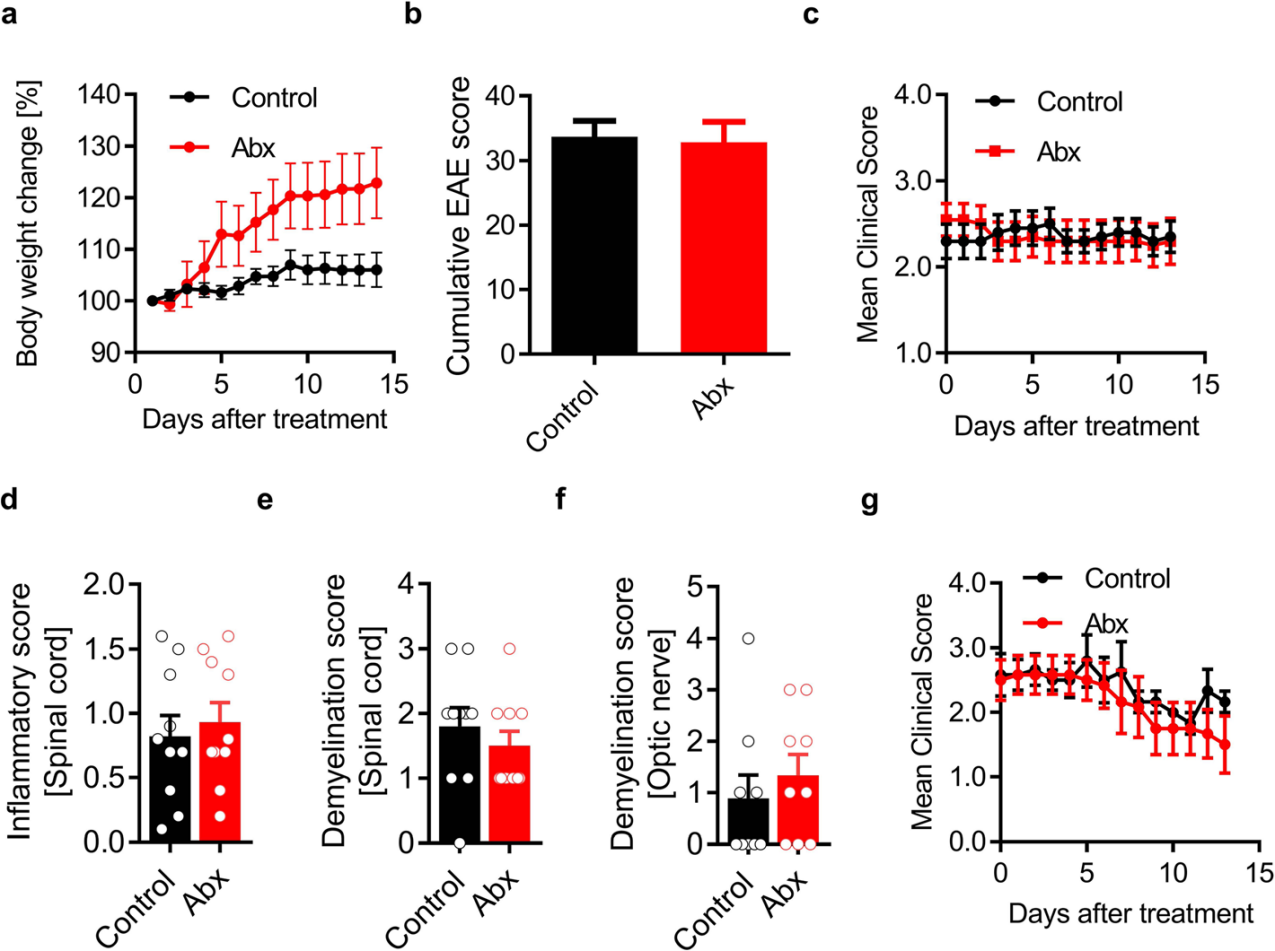
Antibiotic treatment does not affect ongoing CNS autoimmune disease. **a** Bodyweight change in percent compared to the initial weight of EAE-affected OSE mice treated with antibiotics. *n* = 10 per group. **b** Mean cumulative score of EAE-affected OSE mice treated with antibiotics. *n* = 10 per group. **c** Mean clinical score of sick OSE mice treated with antibiotics. *n* = 10 per group. Quantification of the inflammatory score (**d**), demyelination score (**e**) of spinal cord, and demyelination score (**f**) of the optic nerve after 2 weeks of antibiotic treatment. *n* = 10 per group. **g** Mean clinical score of EAE-affected RR SJL/J mice treated with antibiotics. *n* = 6 per group

Having established that antibiotic treatment alters microbiota composition and protects OSE mice from spontaneous EAE development when applied prophylactically, we set out to investigate the impact of antibiotic treatment in clinically established neurological disease. We randomly assigned mice that have developed EAE and gave antibiotics for 2 weeks. Over the course of 2 weeks, we monitored the body weight and their clinical symptoms. The mice that were treated with antibiotics continued to show increased body weight similar to prophylactic antibiotic-treated mice (Fig. 2a). The bacterial load as measured by 16S rRNA gene PCR was also significantly reduced after antibiotic treatment (Supplementary Fig. 3a). However, we did not find significant changes in the disease course during our observation period (Fig. 2b, c). Histological analysis also showed no differences in the inflammatory infiltrates as well as demyelination in the spinal cord and optic nerve (Fig. 2d–f). To rule out model-specific effects, we applied a similar treatment to another spontaneous relapsing-remitting (RR) EAE model on a different genetic background (SJL/J) [13]. Again, antibiotic treatment reduced the bacterial load but had no effect on the disease course (Fig. 2g and Supplementary Fig. 3b). In summary, these data suggest that the microbiota manipulations by antibiotic treatment are not beneficial in a therapeutic regimen.

## Conclusions

The data presented here show that, in the context of CNS autoimmunity, microbiota manipulation is beneficial prior to the disease development but not after established disease. Our data reinforce observations that microbiota manipulations through antibiotics and diet have an impact on inflammatory immune responses that are necessary for the induction of the disease [6, 8–10] while having minimal impact on remyelination processes that are important for the resolution of the disease [15]. Our report provides an important cautionary note that more studies are needed to understand whether microbiota manipulation therapies in clinically established diseases would be beneficial. This is especially important for extra-intestinal organ-specific autoimmune diseases such as MS, where the disease-promoting events may occur elsewhere than the intestine. However, it is possible that the microbiota manipulation in combination with other immunomodulatory drugs will still be useful to mitigate disease symptoms.

## Supplementary information

**Additional file 1 Supplementary Figure 1.** Antibiotics treatment alters microbiota. **(a)** 16 s rRNA gene copies of Enterococci in fecal pellets of antibiotics treated OSE mice measured by quantitative real-time PCR. *n* = 5 per group. (**b - c**) Quantification of Enterococci in fecal pellets by culture on Bile Esculin agar in antibiotics treated OSE mice**.** Serial dilutions of fecal samples collected 6 weeks after the antibiotics treatment were plated on Bile Esculin agar plates. *n* = 4 per group.

**Additional file 2 Supplementary Figure 2.** Representative images of semi-quantitative demyelination scores on cross-sections of the spinal cord and optic nerve stained with Luxol fast blue myelin stain. Spinal cord: SC1: Perivenous and subpial demyelination; SC2: large confluent demyelinated plaques; SC3: extensive demyelination affecting more than half id the spinal cord cross-section. Optic nerve: ON0: no demyelination; ON1: perivenous demyelination; ON2: perivenous and subpial demyelination; ON3: confluent demyelinated plaques; ON4: complete focal demyelination in the optic nerve.

**Additional file 3 Supplementary Figure 3.** Antibiotics treatment in EAE affected mice reduces microbial load. Total 16 s rRNA gene copies in fecal pellets of antibiotics treated OSE mice **(a)** and RR mice **(b)** measured by quantitative real-time PCR. *n* = 4–7 per group. *p < 0.05 (Mann-Whitney test).

## Data Availability

16S rRNA gene amplicon sequencing reads have been deposited at NCBI Sequence Read Archive under BioProject identifier PRJNA605334.

## Abbreviations

CNS: Central nervous system
DNA: Deoxyribonucleic acid
EAE: Experimental autoimmune encephalomyelitis
GF: Germ-free
MS: Multiple sclerosis
OSE: Opticospinal encephalomyelitis
RR: Relapsing-remitting
rRNA: Ribosomal RNA
