# Supplementary figures and images for: Perturbation of gut microbiota decreases susceptibility but does not modulate ongoing autoimmune neurological disease
Source: J Neuroinflammation. 2020 Mar 6;17:79. doi: 10.1186/s12974-020-01766-9 (PMC7060541; doi:10.1186/s12974-020-01766-9)

Supplementary Figure 1

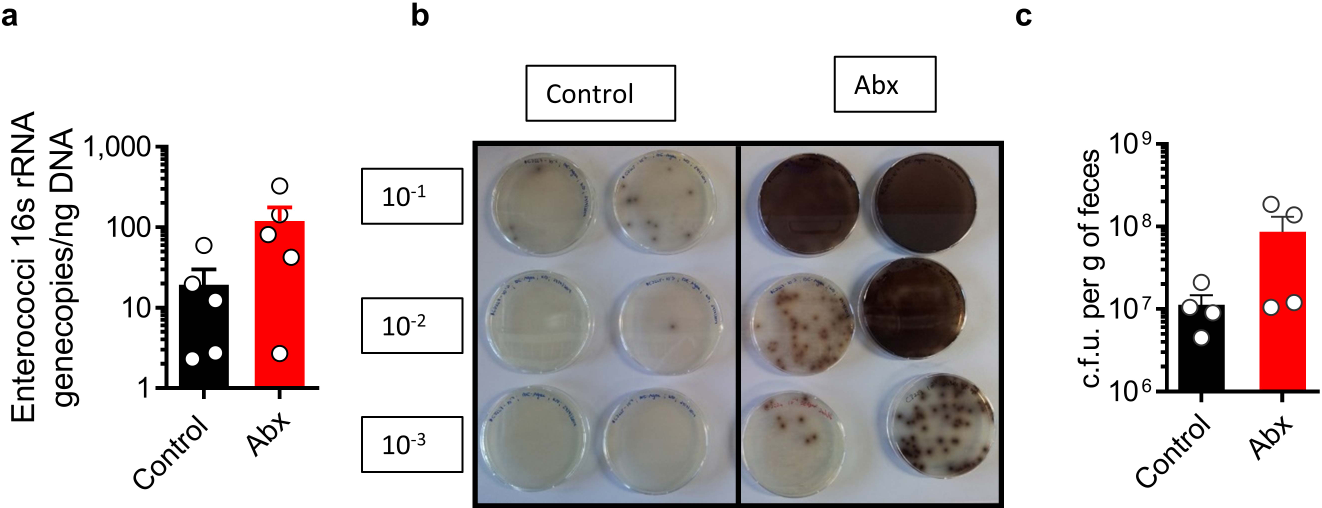

Supplement: Supplementary file 1 — Additional file 1 Supplementary Figure 1. Antibiotics treatment alters microbiota. (a) 16 s rRNA gene copies of Enterococci in fecal pellets of antibiotics treated OSE mice measured by quantitative real-time PCR. n = 5 per group. (b - c) Quantification of Enterococci in fecal pellets by culture on Bile Esculin agar in antibiotics treated OSE mice. Serial dilutions of fecal samples collected 6 weeks after the antibiotics treatment were plated on Bile Esculin agar plates. n = 4 per group. [file 12974_2020_1766_MOESM1_ESM.pdf]

Supplementary Figure 2

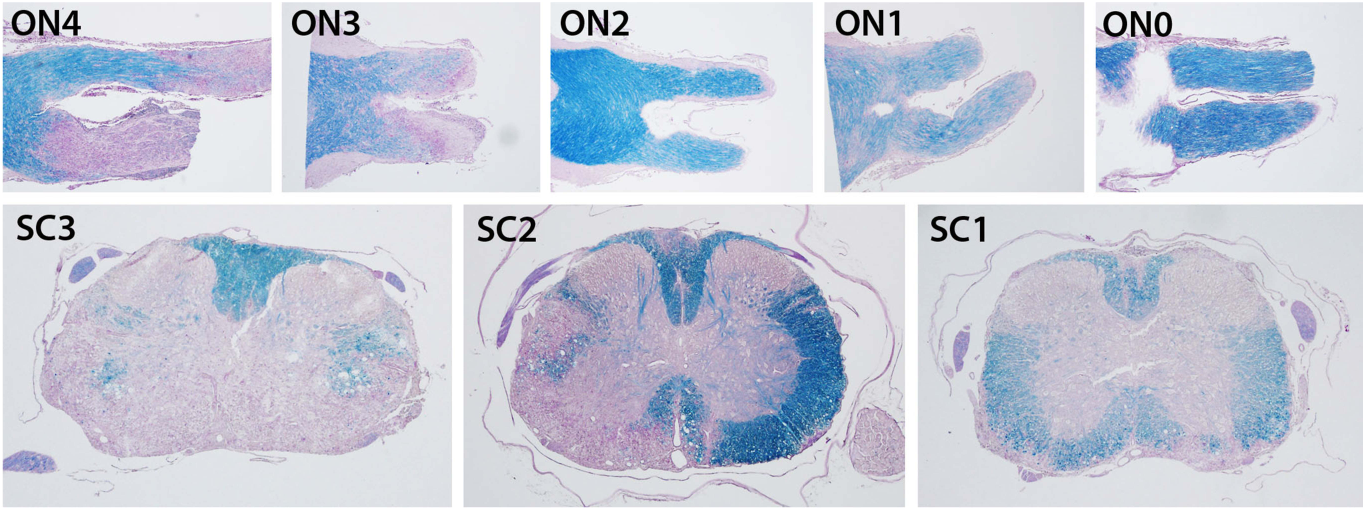

Supplement: Supplementary file 2 — Additional file 2 Supplementary Figure 2. Representative images of semi-quantitative demyelination scores on cross-sections of the spinal cord and optic nerve stained with Luxol fast blue myelin stain. Spinal cord: SC1: Perivenous and subpial demyelination; SC2: large confluent demyelinated plaques; SC3: extensive demyelination affecting more than half id the spinal cord cross-section. Optic nerve: ON0: no demyelination; ON1: perivenous demyelination; ON2: perivenous and subpial demyelination; ON3: confluent demyelinated plaques; ON4: complete focal demyelination in the optic nerve. [file 12974_2020_1766_MOESM2_ESM.pdf]

Supplementary Figure 3

**a** **b**

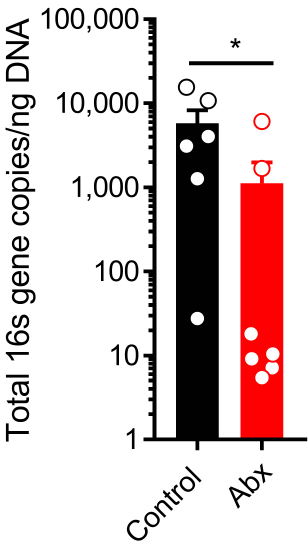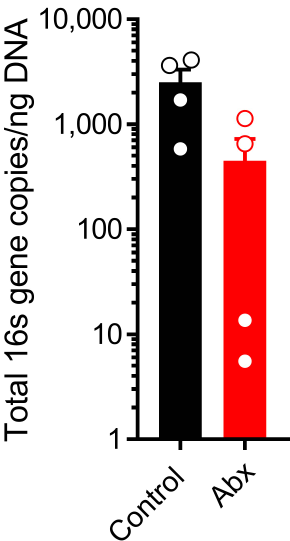

Supplement: Supplementary file 3 — Additional file 3 Supplementary Figure 3. Antibiotics treatment in EAE affected mice reduces microbial load. Total 16 s rRNA gene copies in fecal pellets of antibiotics treated OSE mice (a) and RR mice (b) measured by quantitative real-time PCR. n = 4–7 per group. *p < 0.05 (Mann-Whitney test). [file 12974_2020_1766_MOESM3_ESM.pdf]
